# Favipiravir treatment in Thai children with COVID-19: effectiveness, safety, and modeling drug exposure

**DOI:** 10.2478/abm-2026-0011

**Published:** 2026-04-30

**Authors:** Sujittra Chaisavaneeyakorn, Nopporn Apiwattanakul, Chonnamet Techasaensiri, Nattakarn Tantawarak, Bandit Thinkhamrop, Supattra Rungmaitree, Siriporn Phongjitsiri, Tavitiya Sudjaritruk, Suvaporn Anugulruengkitt, Pope Kosalaraksa, Thirapa Nivesvivat, Puttichart Khantee, Pornumpa Bunjoungmanee, Panit Takkinsatian, Thiraporn Kanjanaphan, Rudiwilai Samakoses, Tim R. Cressey, Henry Pertinez, Andrew Owen, Kulkanya Chokephaibulkit

**Affiliations:** 1 Department of Pediatrics, Faculty of Medicine Ramathibodi Hospital, Mahidol University, Bangkok 10400, Thailand; 2 Department of Pediatrics, Faculty of Medicine, Srinagarind Hospital, Khon Kaen University, Khon Kaen 40002, Thailand; 3 Epidemiology and Biostatistics Section, Faculty of Public Health, Khon Kaen University, Khon Kaen 40002, Thailand; 4 Department of Pediatrics, Faculty of Medicine Siriraj Hospital, Mahidol University, Bangkok 10700, Thailand; 5 Department of Pediatrics, Bhumibol Adulyadej Hospital, Bangkok 10220, Thailand; 6 Department of Pediatrics, Faculty of Medicine, Chiang Mai University, Chiang Mai 50200, Thailand; 7 Department of Pediatrics, Faculty of Medicine, Chulalongkorn University, Bangkok 10330, Thailand; 8 Department of Pediatrics, Phramongkutklao Hospital, Bangkok 10400, Thailand; 9 Department of Pediatrics, Faculty of Medicine, Prince of Songkla University, Songkhla 90110, Thailand; 10 Department of Pediatrics, Faculty of Medicine, Thammasat University, Pathum Thani 12120, Thailand; 11 Department of Pediatrics, Faculty of Medicine, Srinakharinwirot University, Nakhon Nayok 26120, Thailand; 12 Department of Pediatrics, Faculty of Medicine Vajira Hospital, Navamindradhiraj University, Bangkok 10300, Thailand; 13 AMS-PHPT Research Collaboration, Department of Medical Technology, Faculty of Associated Medical Sciences, Chiang Mai University, Chiang Mai 50200, Thailand; 14 Department of Pharmacology and Therapeutics, Centre of Excellence in Long-acting Therapeutics (CELT), University of Liverpool, Liverpool L693BX, Thailand; 15 Siriraj Institute of Clinical Research (SICRES), Faculty of Medicine, Siriraj Hospital, Mahidol University, Bangkok 10700, Thailand

**Keywords:** children, COVID-19, favipiravir, pharmacokinetic modeling, Thailand

## Abstract

**Background:**

Despite widespread favipiravir (FPV) use for coronavirus disease 2019 (COVID-19) in Thailand, pediatric clinical evidence remains limited.

**Objectives:**

This study evaluated FPV’s effectiveness and safety for treating COVID-19 in children and adolescents.

**Methods:**

This multicenter, retrospective, observational cohort study included patients ≤18 years old testing COVID-19 positive between July 2021 and December 2022 across 11 hospitals in Thailand. Patients were divided into those with and without chest X-ray-confirmed pneumonia. Patients who received FPV were treated with 35 mg/kg twice daily on day 1 and 15 mg/kg twice daily on days 2–5. Pharmacokinetic (PK) modeling estimated plasma exposure in children using scaled Thai adult data.

**Results:**

Of 2,999 patients, 68.2% had no pneumonia. FPV recipients (n = 1,886) were mostly <5 years old, had a fever, infected during the Omicron wave, had lower mean cycle threshold (Ct) values of SARS-CoV-2 gene at diagnosis, and hospitalized compared to non-recipients. FPV treatment significantly shortened fever duration and hospitalization in both pneumonia and non-pneumonia patients. For non-pneumonia patients, being <5 years old, and receiving FPV were associated with complete recovery at discharge. For pneumonia patients, a history of preterm birth, pulmonary disease, and ICU admission reduced the likelihood of complete recovery at discharge. No serious adverse events were reported. PK models predicted that FPV plasma trough concentrations in children ≥10 kg were above efficacy targets.

**Conclusions:**

Early FPV treatment shortened fever duration and hospital stays in children and adolescents with COVID-19, promoting clinical recovery in both pneumonia and non-pneumonia cases.

Severe acute respiratory syndrome coronavirus 2 (SARS-CoV-2) has infected nearly 800 million people globally as of December 2024, with a mortality rate of 1% [1]. Children account for 1%–5% of these cases, with a reportedly lower risk of severe coronavirus disease 2019 (COVID-19) and generally milder symptoms than adults [2]. However, some children, particularly those with underlying conditions, may develop severe COVID-19, complications, or even die [2].

According to the United States National Institutes of Health guidelines, remdesivir or ritonavir-boosted nirmatrelvir may be considered for non-hospitalized children and adolescents aged 12–17 who are at high risk of severe COVID-19. Evidence for antiviral use in those under 12 years remains limited. For hospitalized children, only remdesivir is recommended, particularly in severe pneumonia [3]. However, it is only available intravenously and difficult to acquire in many low- and middle-income countries.

Favipiravir (FPV), a nucleotide analog inhibiting viral RNA polymerase, has been registered in China, India, and Russia and approved as an antiviral agent for emergency use and treatment of mild-to-moderate COVID-19 in multiple countries [4–6]. Thailand has approved the use of FPV for COVID-19 since October 2020 [7]. Previous studies in adults with COVID-19 did not demonstrate sufficient evidence regarding the efficacy of FPV treatment in reducing mortality, ventilator support, and hospitalization, regardless of clinical severity [8]. However, a study in Thai adults with mild COVID-19 found that early FPV administration led to faster clinical improvement [9], consistent with a report that FPV treatment, coupled with telemonitoring, effectively prevented clinical worsening and the need for oxygenation in outpatients with mild-to-moderate illness [10].

Clinical data evaluating FPV’s effectiveness in treating COVID-19 in children are limited. One study in Thai children demonstrated a shorter recovery time for all clinical COVID-19 symptoms in patients who received FPV [11]. Given the lack of other oral antiviral treatments for children with mild-to-moderate COVID-19 who are ineligible or lack access to remdesivir, early FPV has been recommended at the discretion of the treating pediatrician.

This study evaluated the effectiveness and safety of FPV in children and adolescents with COVID-19 from a registry cohort in Thailand. Since FPV plasma exposure could not be directly observed, pharmacokinetic (PK) modeling was used to estimate FPV plasma concentrations and compare simulated PK profiles to reported concentration-efficacy targets in this pediatric cohort using current FPV dosing guidelines and PK parameters from allometrically scaled body weights of Thai adults.

## Methods

This study adhered to the standards of the International Council on Harmonisation’s Good Clinical Practice, Declaration of Helsinki, and Belmont Report. Its protocol was approved by the Institutional Ethics Committee at the Faculty of Medicine Ramathibodi Hospital, Mahidol University (COA. MURA2022/127). Written informed consent was waived due to the study’s retrospective design.

### Patients and data collection

This multicenter, retrospective, observational cohort study analyzed data from case record forms of patients ≤18 years who tested positive for COVID-19 (confirmed by nasal swab reverse transcription polymerase chain reaction (RT-PCR) or rapid antigen test for SARS-CoV-2) between July 2021 and December 2022. The study spanned 11 tertiary care hospitals in Thailand, including Ramathibodi Hospital, Phramongkutklao Hospital, Bhumibol Adulyadej Hospital, Siriraj Hospital, Vajira Hospital, and Chulalongkorn University in Bangkok; Prince of Songkla University in Songkla; Thammasat University in Pathum Thani; Srinagarind Hospital in Khon Kaen; and Srinakharinwirot University in Nakhon Nayok. Data review and query management were centralized. It excluded asymptomatic patients, those diagnosed with multisystem inflammatory syndrome in children, patients receiving other antiviral treatment (i.e., ritonavir-boosted nirmatrelvir, remdesivir, or molnupiravir), and cases with missing clinical severity data.

FPV was administered as part of the standard of care based on the Thai National Healthcare Guidelines from 2020 to 2022 and was recommended across all levels of clinical severity throughout the study period [6, 12, 13]. The guideline first became available in October 2020, approximately 9 months after the start of the first COVID-19 wave [6, 12, 14]. FPV originally was recommended for children with pneumonia and was considered for those with risks for severe disease. In the absence of pneumonia, FPV could also be considered for children presented with high fever or significant symptoms. During the Omicron wave, remdesivir became available in April 2022 and prompted a revision to the recommended guidelines; both FPV and remdesivir were to be recommended for children with pneumonia [13].

Patients indicated for FPV initiation (AVIGAN tablets, Fujifilm Toyama Chemical Co., Ltd., 200 mg per tablet) received a dose of 35 mg/kg twice daily (70 mg/kg/d) on day 1, then 15 mg/kg twice daily (30 mg/kg/d) on days 2–5. For younger patients, tablets (or partial tablets) could be dissolved in sweet syrup to facilitate administration and ingested within 30 min. Baseline demographic data, clinical presentations at the time of COVID-19 diagnosis, chest imaging, laboratory investigations, disease severity and complications, treatment, hospitalization, intensive care unit (ICU) admission, intubation and/or oxygen supplementation requirements, and clinical improvement before discharge were recorded.

### Definitions

Complete recovery was defined as the absence of persisting COVID-19 symptoms, while partial recovery was defined as the presence of symptoms at discharge. During the study period, all COVID-19 patients in Thailand were mandated to isolate to control the outbreak. Field hospitals were established for patients who were not severely ill but unable to isolate at home. The length of hospital stay was defined as the total duration of admission to either a hospital or a field hospital. Length of fever was reported from medical records, and defined from maximal collected temperatures ≥37.5°C. The same standard of care was provided in both settings, with ICU cases transferred to hospital facilities. Patients isolating at home or in the community were classified as outpatient cases requiring only isolation to prevent transmission; these were excluded, as their isolation duration did not reflect illness severity.

Thailand experienced its first COVID-19 wave with the ancestral (Wuhan) strain in January 2020, followed by waves of Alpha, Delta, and Omicron variants in April 2021, July 2021, and January 2022, respectively [14]. FPV became available at no charge through the Thai Ministry of Public Health in March 2020 [15].

The World Health Organization’s (WHO) classification system was used to grade COVID-19 severity [16]. The COVID-19 non-pneumonia group in this study included those with mild COVID-19 (defined by an upper respiratory tract infection or other mild symptoms without evidence of pneumonia) [17, 18]. The COVID-19 pneumonia group included patients with moderate disease (defined as non-severe pneumonia confirmed by abnormal chest radiological findings), severe disease (defined as pneumonia with pulse oximetry <90%, severe respiratory distress, inability to breastfeed or drink, lethargy or unconsciousness, and/or convulsions), and critical disease (defined as acute respiratory distress syndrome, sepsis, or septic shock) [17, 18]. Laboratory investigations and cutoff ranges were specific to the patient’s age. Lymphopenia was defined as an absolute lymphocyte counts below the thresholds: <2,000 cells/mm^3^ for infants aged 0-2 weeks, <2,500 for 1–6 months, <4,000 for 6 months to 1 year, <3,000 for 1-2 years, <2,000 for 2–4 years, <1,500 for 4–10 years, <1,200 for 10–16 years, and <1,000 for individuals >16 years [19]. Aspartate aminotransferase (AST) were defined to be elevated if they exceeded the referenced ranges: 7–150 U/L for infants aged 0–10 d, 9–80 U/L for 10 d to 24 months, and 13–35 U/L for female and 15–40 U/L for male patients >24 months [19].

### PK modeling and simulation of plasma FPV concentrations in children

The authors have recently published a one-compartment PK model with first-order absorption that describes plasma FPV concentrations in 8 Thai adults with mild COVID-19 [20]. Model PK parameters were adjusted through allometric scaling [21] to body weights of 3 kg, 5 kg, 8 kg, 10 kg, 20 kg, or 35 kg to estimate FPV plasma exposure in children using the same dosing regimen as this study (35 mg/kg twice daily [70 mg/kg/d] on day 1, then 15 mg/kg twice daily [30 mg/kg/d] on day 2–5).

FPV clearance (CL), volume of distribution (Vd), and absorption rate constant (KA) values from Thai adults were scaled using the following equations [21], with fixed allometric exponents of 0.75 (1), 1.0 (2), and –0.25 (3), respectively, relative to the mean adult body weight of 60 kg in the Thai adult PK study, to calculate typical PK model parameters for children of the body weights evaluated:

1 $$\begin{array}{ll} {\text{Estimated CL}}_{\text{child}} & =\left({\text{CL}}_{\text{apualt}}\right)\;\;\times \;\;{\left({\text{weight}}_{\text{child}}/60\right)}^{0.75}\\ & =(1\;\text{L}/\text{h})\;\;\times \;\;{\left({\text{weight}}_{\text{child}}/60\right)}^{0.75} \end{array}$$

2 $$\begin{array}{ll} {\text{Estimated Vd}}_{\text{child}} & =\left({\text{Vd}}_{\text{adult}}\right)\;\;\times \;\;{\left({\text{ weight}}_{\text{child}}/60\right)}^{1.0}\\ & =(19.5\;\text{L})\;\;\times \;\;{\left({\text{ weight}}_{\text{child}}/60\right)}^{1.0} \end{array}$$

3 $$\begin{array}{ll} {\text{Estimated K}}_{\text{A child}} & =\left({\text{K}}_{\text{A adult}}\right)\times {\left({\text{ weight}}_{\text{child}}/60\right)}^{-0.25}\\ & =\left(3.5\;{\text{h}}^{-1}\right)\times {\left({\text{ weight}}_{\text{child}}/60\right)}^{-0.25} \end{array}$$

Plasma PK profile simulations were performed in R (v4.3) [22], and plasma exposures presented in relation to FPV’s 90% effective concentration (EC_90_) against SARS-CoV-2 of 159 μM, as determined in vitro using Vero-E6 cell lines [23] and the trough concentration (*C*_trough_) in hamsters using a FPV regimen that demonstrated efficacy in that species [24].

### Statistical analysis

Categorical data were reported as frequency and percentages. Chi-square or Fisher exact tests were used to compare categorical variables. Quantitative data were reported as mean (standard deviation [SD]) or median (interquartile range [IQR]), depending on the data distribution. Mann–Whitney *U* or Student’s *t*-tests were used to compare continuous variables. Potential factors associated with complete clinical recovery at hospital discharge were analyzed using logistic regression. Factors associated with duration of fever were analyzed using linear regression analysis. Factors with *P* < 0.10 in the univariable analysis were included in multivariable analyses. Statistical analysis was performed using STATA (v18.0, StataCorp LLC). Missing data were excluded from the final analyses.

## Results

**Figure 1.**
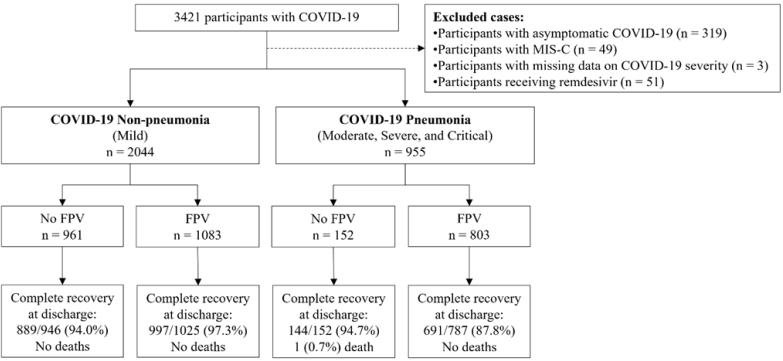
Patient flowchart. COVID-19, coronavirus disease 2019; FPV, favipiravir; MIS-C, multisystem inflammatory syndrome in children.

Of the 3,421 patients with COVID-19, 87.7% (n = 2,999/3,421) were included in the analysis after screening (Figure 1). Of the 2,999 included patients, 68.2% (n = 2,044/2,999), 28.5% (n = 855/2,999), 2.9% (n = 86/2,999), and 0.5% (n = 14/2,999) had a mild, moderate, severe, and critical disease severity, respectively (Table 1). Patients with documented SARS-CoV-2 diagnosis underwent RT-PCR testing with 100% (n = 909) in the pneumonia and 99.7% (n = 1,814/1,820) in the non-pneumonia groups (Table 1). Rapid antigen testing was performed in 95.8% (n = 158/165) in the pneumonia group and 96.1% (n = 421/438) in the nonpneumonia group (Table 1). Of the limited documentation for COVID-19 vaccinations, 1.2% (n = 23/1,925) were vaccinated once, 1.4% (n = 27/1,925) were vaccinated for 2 doses, and 0.2% (n = 3/1,925) were vaccinated for 3 doses (data not included in table).

**Table 1.** Demographics, clinical characteristics, and outcomes of children and adolescents with and without COVID-19 pneumonia and treatment with FPV.

Demographics and clinical characteristics	COVID-19 non-pneumonia (n = 2,044)			COVID-19 pneumonia (n = 955)		
	No FPV (n = 961)	FPV (n = 1,083)	*P*	No FPV (n = 152)	FPV (n = 803)	*P*
Age (years)						
<5	185 (19.3)	578 (53.4)	<0.001	52 (34.2)	459 (57.2)	<0.001
5–18	776 (80.7)	503 (46.5)		100 (65.8)	344 (42.8)	
Biological sex (male)	n = 961	n = 1,081		n = 152	n = 803	
	472 (49.1)	588 (54.4)	0.017	79 (52.0)	430 (53.6)	0.211
Underlying conditions	n = 863	n = 981		n = 148	n = 787	
	109 (12.6)	290 (29.6)	<0.001	25 (16.9)	186 (23.6)	0.072
Cardiovascular disease	n = 130	n = 335		n = 34	n = 192	
	7 (5.4)	30 (9.2)	0.181	1 (2.9)	27 (14.1)	0.070
Gastrointestinal disease	n = 127	n = 320		n = 33	n = 190	
	0 (0.0)	1 (0.3)	1.000	0 (0.0)	0 (0.0)	NA
Genetic disease	n = 127	n = 321		n = 34	n = 190	
	0 (0.0)	9 (2.8)	0.066	1 (3.0)	8 (4.2)	1.000
Hematologic malignancy	n = 127	n = 321		n = 33	n = 191	
	1 (0.8)	16 (5.1)	0.050	1 (3.0)	5 (2.6)	1.000
History of preterm birth	n = 127	n = 320		n = 33	n = 193	
	0 (0.0)	11 (3.4)	0.039	0 (0.0)	15 (7.8)	0.136
Neurological disease	n = 128	n = 323		n = 34	n = 192	
	4 (3.1)	11 (3.4)	1.000	2 (5.9)	17 (8.9)	0.746
Pulmonary disease	n = 139	n = 331		n = 35	n = 201	
	44 (31.7)	80 (24.2)	0.093	6 (17.1)	56 (27.9)	0.184
Obesity	n = 128	n = 327		n = 33	n = 193	0.214
	9 (7.0)	33 (10.1)	0.311	1 (3.0)	21 (10.9)	
Renal disease	n = 127	n = 321		n = 33	n = 190	
	0 (0.0)	3 (0.9)	0.562	0 (0.0)	2 (1.1)	1.000
Rheumatological disease	n = 127	n = 320		n = 33	n = 190	
	0 (0.0)	3 (0.9)	0.562	0 (0.0)	1 (0.5)	1.000
Solid tumor	n = 127	n = 321		n = 33	n = 191	
	0 (0.0)	4 (1.3)	0.581	0 (0.0)	6 (3.1)	0.596
Transplantation	n = 127	n = 320		n = 33	n = 191	
	0 (0.0)	3 (0.9)	0.562	0 (0.0)	2 (1.1)	1.000
COVID-19 vaccination	n = 555	n = 619		n = 134	n = 617	
	14 (2.5)	28 (4.5)	0.065	1 (0.8)	10 (1.6)	0.699
Predominant strain during infection^†^	n = 961	n = 1,083		n = 152	n = 803	
Pre-delta/omicron	314 (32.7)	62 (5.7)	<0.001	71 (46.7)	134 (16.7)	<0.001
Delta	477 (49.6)	319 (29.5)		65 (42.8)	317 (39.5)	
Omicron	170 (17.7)	702 (64.8)		16 (10.5)	352 (43.8)	
SARS-CoV-2 Diagnosis						
Rapid antigen test	144 (98.0)	277 (95.2)	0.196	12 (92.3)	146 (96.1)	0.443
	n = 147	n = 291		n = 13	n = 152	
Waves						
Pre-delta/omicron	1 (100)	1 (100)	NA	2 (100)	2 (100)	NA
Delta	52 (98.1)	40 (95.2)	0.582	3 (100)	32 (100)	NA
Omicron	91 (97.9)	236 (95.2)	0.366	7 (88.0)	112 (94.9)	0.376
Sites						
Non-hospital	101 (99.0)	49 (98.0)	0.551	3 (100)	14 (100)	NA
Hospital	43 (95.6)	228 (94.6)	1.000	9 (90.0)	132 (95.7)	0.394
RT-PCR	841 (99.8)	973 (99.6)	0.692	149 (100)	760 (100)	NA
	n = 843	n = 977		n = 149	n = 760	
Waves						
Pre-delta/omicron	298 (99.3)	58 (100)	1.000	70 (100)	130 (100)	NA
Delta	447 (100)	306 (100)	NA	64 (100)	304 (100)	NA
Omicron	96 (100)	609 (99.4)	1.000	15 (100)	326 (100)	NA
Sites						
Non-hospital	477 (99.6)	106 (100)	1.000	68 (100)	151 (100)	NA
Hospital	364 (100)	867 (99.5)	0.326	81 (100)	609 (100)	NA
Ct of SARS-CoV-2 RT-PCR at diagnosis, mean ± SD						
N gene	n = 628	n = 773		n = 124	n = 629	
	24.2 ± 6.2	20.8 ± 5.8	<0.001	24.3 ± 5.6	22.4 ± 6.1	0.001
*ORF* gene	n = 399	n = 607		n = 100	n = 531	
	25.2 ± 6.9	22.0 ± 5.9	<0.001	24.0 ± 5.6	22.3 ± 5.9	0.008
Clinical severity	n = 961	n = 1,083		n = 152	n = 803	
Mild	961 (100.0)	1,083 (100.0)	NA	-	-	0.007
Moderate	-	-		147 (96.7)	708 (88.2)	
Severe	-	-		4 (2.6)	82 (10.2)	
Critical illness	-	-		1 (0.7)	13 (1.6)	
*Outcomes of treatment*						
Duration (days) of FPV treatment, median (IQR)		n = 1,071			n = 801	
	-	4 (4–5)	NA	-	4 (4–5)	NA
Duration (days) between diagnosis and FPV initiation, median (IQR)		n = 1,077			n = 802	
	-	1 (0–1)	NA	-	1 (0–2)	NA
Duration (days) between symptom onset to FPV initiation, median (IQR)		n = 1,046			n = 673	
	-	1 (1–3)	NA	-	2 (1–3)	NA
Received antibiotics	5 (0.5)	44 (4.1)	<0.001	7 (4.6)	89 (11.1)	0.015
Received corticosteroids	3 (0.3)	25 (2.3)	<0.001	2 (1.3)	69 (8.6)	0.002
Received oxygenation	0 (0.0)	8 (0.7)	0.008	0 (0.0)	98 (12.2)	<0.001
Isolation						
Home/community	296 (30.8)	77 (7.1)	<0.001	21 (13.8)	74 (9.2)	<0.001
Field hospital	263 (27.4)	61 (5.6)		49 (32.3)	83 (10.3)	
Hospital	402 (41.8)	945 (87.3)		82 (54.0)	646 (80.5)	
Duration of fever (days), median (IQR)	n = 55 8 (5–14)	n = 590 6 (5–8)	0.012	n = 23 14 (10–14)	n = 382 7 (5–9)	<0.001
Condition at discharge	n = 946	n = 1,025				
Complete recovery	889 (94.0)	997 (97.3)	<0.001	144 (94.7)	691 (87.8)	0.013
Partial recovery	57 (6.0)	28 (2.7)		7 (4.6)	96 (12.2)	
Death	0 (0.0)	0 (0.0)		1 (0.7)	0 (0.0)	
Length of hospital admission, median (IQR)	n = 663 10 (7–11)	n = 1,002 5 (3–8)	<0.001	n = 130 11 (8–13)	n = 720 8 (5–11)	<0.001
Hospital	9 (6–10) n = 401	5 (3–8) n = 941	<0.001	12 (8–13) n = 81	8 (4–10) n = 638	<0.001
Field hospital	10 (8–12) n = 262	11 (9–12) n = 61	0.074	10 (8–12) n = 49	11 (9–13) n = 82	0.074

1 Mann-Whitney *U* or Student’s *t*-tests were used to compare continuous variables. Data are presented as n (%) unless stated otherwise.

† The ancestral Wuhan strain and alpha variant were the predominant circulating strains during the first to third waves (Pre-delta/omicron). Delta and omicron variants were the predominant circulating strains during the fourth and fifth waves, respectively.

1 COVID-19, coronavirus disease 2019; Ct, cycle threshold; FPV, favipiravir; IQR, interquartile range; NA, not applicable; N gene, nucleocapsid gene; *ORF* gene, open reading frame gene; RT-PCR, reverse transcription polymerase chain reaction; SARS-CoV-2, severe acute respiratory syndrome coronavirus 2; SD, standard deviation.

## Patients without pneumonia

About half (n = 1,083/2,044, 53.0%) of non-pneumonia COVID-19 patients received FPV (Table 1). A significantly higher proportion of these who received FPV were <5 years old (53.4% vs. 19.3%, *P* < 0.001), male (54.4% vs. 49.1%, *P =* 0.017), had underlying conditions (29.6% vs. 12.6%, *P* < 0.001), infected during the Omicron predominant period (64.8% vs. 17.7%, *P* < 0.001), on antibiotics (4.1% vs. 0.5%, *P* < 0.001) or corticosteroids (2.3% vs. 0.3%, *P* < 0.001), and hospitalized (87.3% vs. 41.8%, *P* 0.001) (Table 1). The mean baseline cycle threshold (Ct) of the SARS-CoV-2 nucleocapsid (N) (20.8 vs. 24.2, *P* < 0.001) and open reading frame (ORF) (22.0 vs. 25.2, *P* < 0.001) genes, quantified by RT-PCR, were lower in those who received FPV than those who did not. Patients who received FPV also had a significantly higher frequency of fever, diarrhea, nausea/vomiting, and febrile seizures (*P* < 0.001 for all symptoms) as well as a lower frequency of coughing (*P* < 0.001) and nasal discharge (*P =* 0.002) compared to those who did not receive FPV (Supplementary Table 1).

From available data, patients who received FPV had a shorter median duration of fever (6 d vs. 8 d, *P =* 0.012) and hospitalization (5 d vs. 10 d, *P* < 0.001) compared with those who did not receive FPV. When adjusting for care settings, this reduction in hospitalization remained significant among hospital admitted patients (5 d vs. 9 d, *P* < 0.001). Those that received FPV also reported a higher rate of complete recovery at discharge (97.3% vs. 94%, *P* < 0.001) than those who did not. The data are shown in Table 1.

## Patients with pneumonia

Most (n = 803/955, 84.1%) of the COVID-19 pneumonia patients received FPV. A significantly higher proportion of those who received FPV according to the national guidelines were <5 years old (57.2% vs. 34.2%, *P* < 0.001), infected during the Omicron wave (43.8% vs. 10.5%, *P* < 0.001), on antibiotics (11.1% vs. 4.6%, *P =* 0.015) or steroids (8.6% vs. 1.3%, *P =* 0.002), hospitalized (80.5% vs. 54.0%, *P* < 0.001), and had severe or critical illness (11.8% vs. 3.3%, *P =* 0.007). The mean baseline Ct of the SARS-CoV-2 N (22.4 vs. 24.3, *P =* 0.001) and ORF (22.3 vs. 24.0, *P =* 0.008) genes, quantified by RT-PCR, were also lower in those receiving than not receiving FPV (Table 1). A significantly higher frequency of fever (*P* < 0.001) and nausea/vomiting (*P =* 0.026) were seen in patients who received FPV (Supplementary Table 1).

Similar to the available data for patients without pneumonia, the median duration of fever (7 d vs. 14 d, *P* < 0.001) and hospitalization (8 d vs. 11 d, *P* < 0.001) were significantly shorter in patients with pneumonia who received FPV However, only hospital-administered patients reported significantly reduced median hospitalization duration (8 d vs. 12 d, *P* < 0.001) when adjusted for care settings (Table 1). A lower rate of complete recovery at discharge was observed in patients who received FPV (87.8% vs. 94.7%, *P =* 0.013) (Table 1).

## Factors associated with complete recovery at discharge

Being <5 years old {adjusted odds ratio (aOR) 1.9 (95% confidence interval [CI] 1.1, 3.3), *P =* 0.030} and receiving FPV (aOR 1.9 [95% CI 1.2, 3.1], *P =* 0.010) were independently associated with complete recovery at discharge for COVID-19 non-pneumonia patients. Contracting SARS-CoV-2 during the Omicron-predominant period was also associated with a significantly higher likelihood of complete recovery at discharge in univariate analysis (odds ratio [OR] 3.2 [95% CI 1.7, 5.9], *P* < 0.001) but was not significant in multivariate analysis (Table 2).

**Table 2.** Potential factors for predicting clinical recovery at discharge of patients with and without COVID-19 pneumonia

Factors	COVID-19 non-pneumonia				COVID-19 pneumonia			
	OR (95% CI)	*P*	aOR (95% CI)	*P*	OR (95% CI)	*P*	aOR (95% CI)	*P*
Age (years)								
<5	2.4 (1.4, 4.1)	0.002	1.9 (1.1, 3.3)	0.030				
5–18	Reference		Reference					
Underlying disease								
History of preterm birth					0.3 (0.1, 0.9)	0.033	0.2 (0.1, 0.7)	0.011
Pulmonary disease					0.4 (0.2, 0.9)	0.031	0.4 (0.2, 0.9)	0.028
Predominant strain during infection†								
Pre-delta/omicron	Reference				Reference			
Delta	1.3 (0.8, 2.1)	0.360			0.2 (0.1, 0.5)	<0.001		
Omicron	3.2 (1.7, 5.9)	<0.001			0.3 (0.1, 0.7)	0.003		
Treatment								
FPV	2.3 (1.4, 3.6)	<0.001	1.9 (1.2, 3.1)	0.010	0.4 (0.2, 0.8)	0.016		
ICU admission					0.2 (0.1, 0.5)	<0.001	0.1 (0.1, 0.4)	0.001

1 Factors with *P* < 0.10 in the univariable analysis were included in multivariable analyses.

† The ancestral Wuhan strain and alpha variant were the predominant circulating strains during the first to third waves (Pre-delta/omicron). Delta and omicron variants were the predominant circulating strains during the fourth and fifth waves, respectively.

1 aOR, adjusted odds ratio; CI, confidence interval; COVID-19, coronavirus disease 2019; FPV, favipiravir; ICU, intensive care unit; OR, odds ratio.

A history of preterm birth (aOR 0.2 [95% CI 0.1, 0.7], *P =* 0.011), pulmonary disease (aOR 0.4 [95% CI 0.2, 0.9], *P =* 0.028), and ICU admission (aOR 0.1 [95% CI 0.1, 0.4], *P =* 0.001) were associated with a lower likelihood of complete recovery at discharge for COVID-19 pneumonia patients (Table 2). Receiving FPV (OR 0.4 [95% CI 0.2, 0.8], *P =* 0.016), contracting SARS-CoV-2 during the Delta-predominant period (OR 0.2 [95% CI 0.1, 0.5], *P* < 0.001), and contracting SARS-CoV-2 during the Omicron-predominant period (OR 0.3 [95% CI 0.1, 0.7], *P =* 0.003) were associated with a lower rate of complete recovery in univariate analysis; however, they were not statistically significant in multivariable analysis (Table 2). Baseline characteristics by recovery status are summarized in Supplementary Table 2.

In a sub-analysis of patients with and without pneumonia who had fever at diagnosis (n = 2,076/2,988, 69.5%), presence of pneumonia was significantly associated with longer durations of fever (β 0.49 [95% CI 0.07–0.90], *P =* 0.022), whereas contracting SARS-CoV-2 during the Omicron predominant period (β -3.02 [95% CI –3.85, –2.19], *P* < 0.001) and receiving FPV (*β –*2.66 [95% CI –3.44, –1.88], *P* < 0.001) were significantly associated with shorter fever durations (Table 3).

**Table 3.** Factors associated with fever duration in COVID-19 participants who had a fever at baseline (n = 2,076 participants)

Variables	Coefficient(d)	(95% CI)	*P*	Coefficient(d)	(95% CI)	*P*
Age (years)						
<5	Reference					
≥5	1.1	(0.74, 1.55)	<0.001			
Biological sex						
Male	Reference					
Female	0.08	(–0.33, 0.49)	0.704			
Underlying disease	–0.03	(–0.50, 0.43)	0.890			
History of COVID-19 vaccination	–1.12	(–2.39, 0.14)	0.082			
Predominant strain during infection^†^						
Pre-delta/pre-omicron	Reference					
Delta	–0.38	(–1.21,0.46)	0.377			
Omicron	–3.33	(–4.12, –2.55)	<0.001	–3.02	(–3.85, –2.19)	<0.001
Clinical severity						
Non-pneumonia	Reference					
Pneumonia	0.73	(0.32, 1.15)	0.001	0.49	(0.07, 0.90)	0.022
Treatment						
FPV	–3.21	(–3.99, –2.44)	<0.001	–2.66	(–3.44, –1.88)	<0.001
Antibiotics	1.1	(0.30, 1.92)	0.008			
Time from COVID-19 diagnosis until FPV	0.94	(0.80, 1.08)	<0.001			
administration (d)						

1 Factors with *P* < 0.10 in the univariable analysis were included in multivariable analyses.

† The ancestral Wuhan strain and alpha variant were the predominant circulating strains during the first to third waves (Pre-delta/omicron). Delta and omicron variants were the predominant circulating strains during the fourth and fifth waves, respectively.

1 CI, confidence interval; COVID-19, coronavirus disease 2019; FPV, favipiravir; RT-PCR, reverse transcription polymerase chain reaction.

## Laboratory results and adverse events (AEs)

In COVID-19 non-pneumonia patients who received FPV, the frequency of lymphopenia was lower (43.3% vs. 54.8%, *P =* 0.036), and elevated AST (29.3% vs. 15.6%, *P =* 0.011) as well as median peak AST levels (37 vs. 30, *P =* 0.001) were higher at treatment completion compared to initiation (Supplementary Table 3). In COVID-19 pneumonia patients, complete blood count and liver function tests were not statistically different between the initiation and completion of FPV treatment (Supplementary Table 3). No patients were discontinued due to available AEs documentation.

## FPV exposure from modeling using the recommended dosing

**Figure 2.**
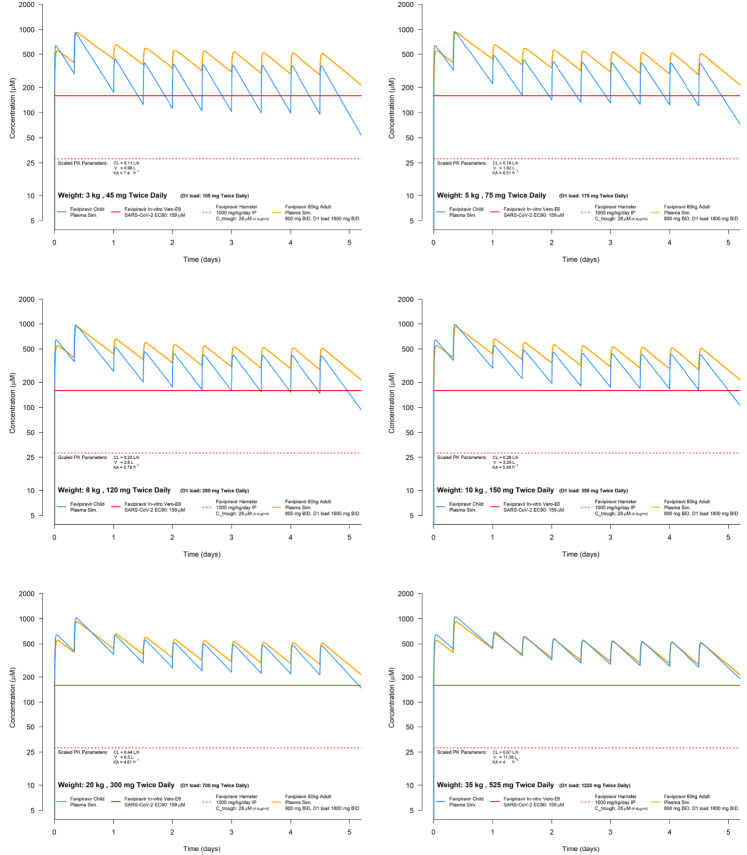
Simulations of FPV plasma PK in Thai children and adolescents weighing 3 kg, 5 kg, 8 kg, 10 kg, 20 kg, and 35 kg. PK parameter values at these weights were derived from allometric scaling of plasma PK observed in Thai adults weighing 60 kg. BID, twice daily; CL, clearance; Ctrough, trough concentration; D, day; EC90, 90% effective concentration; FPV, favipiravir; IP, investigational product; KA, absorption rate constant; PK, pharmacokinetics; Vd, volume of distribution.

Simulated FPV plasma concentration profiles for pediatric patients, using the recommended loading and maintenance doses, are shown in Figure 2. Exposures in patients were anticipated to be generally lower than typical in Thai adults with a body weight of 60 kg, who were treated with 1,800/800 mg FPV twice daily loading/maintenance doses. Children of all weights examined were predicted to achieve plasma exposures higher than the levels known to be efficacious in hamster infection models (28 μM). However, only children weighing ≥10 kg were expected to consistently achieve plasma *C* trough above the in vitro FPV EC90 for the 5 d of treatment. Smaller, younger children (e.g., 3 kg or 5 kg) were expected to have *C*_trough_ just below 159 μM by Day 2 of treatment.

## Discussion

This study investigated the clinical benefits and AEs of FPV in children and adolescents with COVID-19. From the available data, clinical benefits of FPV supports the shortening of fever duration and hospital stay, irrespective of the disease’s clinical severity. These findings also suggest that in patients without pneumonia, younger age (<5 years old) and FPV treatment were associated with a higher likelihood of complete recovery at discharge. In contrast, among pneumonia patients, FPV was not associated with complete recovery, while prematurity, pulmonary diseases, and ICU admission reduced the likelihood of complete recovery. FPV was safe, well tolerated, and had only minimally elevated transaminase levels. The administered pediatric FPV dose was predicted to achieve target FPV plasma *C*_trough_ in children ≥10 kg based on PK modeling and simulation.

These findings regarding which patients were more likely to receive FPV across both pneumonia and non-pneumonia groups reflect the bias of using FPV in sicker patients. This aligns with the Thai National Treatment Guidelines for COVID-19, which recommend FPV for symptomatic children without pneumonia but with risk factors for severe disease. For patients with pneumonia or severe disease, remdesivir is recommended while FPV is an optional treatment when remdesivir is not accessible. Recently, FPV is no longer recommended for children with pneumonia due to the improved availability of remdesivir, but it remains an oral treatment option for those without pneumonia who are at risk of developing it. When using FPV, the guidelines emphasize early initiation of treatment [12, 13].

Compared to those who did not receive FPV, these findings indicate that FPV treatment was associated with a reduced duration of fever and length of hospital admission. This aligns with previous literature in adults, which demonstrated an association between early FPV treatment and reduced fever duration [25, 26] as well as its clinical benefits [27–30], with one study reporting a median time to sustained clinical improvement of 2 d compared to 14 d in the controlled group [9]. Some studies found that FPV treatment did not provide clinical benefits or improve virologic clearance in adult patients with mild-to-moderate COVID-19 [31–33], while other studies revealed better viral clearance and clinical outcomes in patients who received FPV compared to control patients [28, 29, 34, 35]. Differences in treatment initiation times, insufficient sample sizes for the desirable endpoints, and variability in drug exposure across ethnic groups may explain reported inconsistencies in efficacy. Despite these inconsistencies, this study and others consistently report FPV as safe and well tolerated [28, 36, 37]. Current recommended FPV doses in Thailand achieve target plasma concentrations in Thai adults, with higher exposures than those in Chinese, Japanese, and Turkish populations [20]. There are limited data in children, with only a few reports of safety profiles [9, 27, 38, 39]. To the authors’ knowledge, this is the largest report of FPV treatment for COVID-19 in children.

In addition, data from this study indicated that FPV treatment in mild cases was associated with a lower likelihood of persistent COVID-19 symptoms at discharge compared to those who did not receive FPV. These findings support the potential benefits for FPV use in mild and moderate cases of COVID-19 in children and adolescents. Host immunity and risk conditions are additional important factors influencing disease severity and likelihood of patients’ full recovery. As found in multivariable analysis, underlying diseases and admission to ICU were independent factors related to a reduced likelihood of complete recovery at discharge for those with pneumonia. Those with pneumonia who received FPV treatment were younger and relatively more unwell, with a greater frequency of fever and lower baseline Ct values, which could explain the lower proportion of complete recovery among these patients.

This study had some limitations. First, as this was a retrospective study, the baseline characteristics (i.e., underlying disease, clinical severity, and fever duration) of patients with and without FPV were dependent on existing documentation or were not balanced. However, this limitation was biased toward sicker patients in the FPV group, thereby supporting the true clinical benefit. Second, although discharge criteria were not predefined across sites, they were consistently applied between the FPV and non-FPV groups within each site and based on local routine clinical practices and timepoint. Third, there were limited data regarding laboratory investigations and standardization during patients’ hospital stays (e.g., viral clearance, sequencing data for the SARS-CoV-2 variants, and detailed PCR testing information), and thus limiting the use of these data in multivariable analysis. Toxicity laboratory results were also limited, with few paired data points available; as such, uric acid-–he most reported laboratory abnormality associated with FPV–was not measured in most pediatric patients. Availability of AE reporting was additionally limited to previously documented records, affecting reports of additional AEs of interest (e.g., parental concerns, corneal/scleral discoloration, or rash). Fourth, only a few children in this study were vaccinated. This could limit the generalizability of these findings to the current vaccinated population. Fifth, this study did not include any long-term follow-ups. One strength of this study was its multicenter design, which included data from 11 hospitals across Thailand, spanning from the start of the COVID-19 pandemic to the Omicron wave. To the authors’ knowledge, the PK modeling data in children and adolescents is the first ever reported and confirmed the optimal exposure with the current dosing recommended in the National Guidelines. However, predictions in small children rely on allometric assumptions, which may not fully apply to very young populations.

## Conclusions

From the available data, the study suggests that early initiated FPV treatment in children and adolescents with COVID-19 has clinical benefits, shortening the durations of fever and hospital stay and assisting recovery in those with and without pneumonia. FPV was safe and well-tolerated. Optimal PK exposure in Thai patients may explain regional differences in FPV effectiveness, warranting further investigation. Due to the limited options for oral antiviral treatment in children, FPV may be considered in children and adolescents with mild COVID-19 who have risk factors for severe disease. Future randomized controlled trials should study the efficacy and effectiveness of FPV as well as optimal PK exposure in Thai patients.

## Figures and Tables

**Supplementary Table 1.** Clinical manifestations of COVID-19 patients with and without pneumonia and favipiravir treatment at the time of diagnosis

Clinical manifestations	COVID-19 non-pneumonia			COVID-19 pneumonia		
	No FPV n = 961	FPV n = 1083	*P*	No FPV n = 152	FPV n = 803	*P*
Fever	497 (52.0) n = 956	912 (84.5) n = 1079	<0.001	53 (34.9) n = 152	614 (76.7) n = 801	<0.001
Chills	4 (0.4) n = 956	8 (0.7) n = 1079	0.397	1 (0.7) n = 152	2 (0.3) n = 801	0.804
Conjunctivitis	3 (2.1) n = 140	2 (1.4) n = 147	0.678	0 (0.0) n = 20	2 (0.9) n = 233	1.000
Nasal discharge	450 (48.7) n = 925	446 (41.9) n = 1065	0.002	69 (59.0) n = 117	357 (46.7) n = 765	0.013
Dyspnea	13 (1.4) n = 919	20 (1.9) n = 1060	0.413	5 (4.4) n = 115	76 (10.0) n = 763	0.053
Cough	654 (70.3) n = 930	661 (61.9) n = 1068	<0.001	90 (77.6) n = 116	542 (70.6) n = 768	0.119
Abdominal pain	11 (1.2) n = 920	21 (2.0) n = 1060	0.167	5 (4.4) n = 115	5 (0.7) n =763	0.005
Diarrhea	78 (8.5) n = 921	142 (13.4) n = 1062	<0.001	13 (11.3) n = 115	102 (13.3) n = 765	0.547
Nausea/vomiting	38 (4.1) n = 920	207 (19.5) n = 1062	<0.001	9 (7.8) n = 115	120 (15.7) n = 763	0.026
Rash	5 (0.5) n = 919	7 (0.7) n = 1060	0.740	0 (0) n = 115	3 (0.4) n = 763	1.000
Febrile seizure	0 (0.0) n = 919	18 (1.7) n = 1060	<0.001	2 (1.7) n = 115	38 (5.0) n = 763	0.120

1 Mann–Whitney *U* or Student’s *t*-tests were used to compare continuous variables. Data are presented as n (%) unless stated otherwise.

1 COVID-19, coronavirus disease 2019; FPV, favipiravir.

**Supplementary Table 2.** Demographic and clinical characteristics of COVID-19 participants with and without pneumonia and partial or complete clinical recovery at discharge.

Potential factors	COVID-19 non-pneumonia			COVID-19 pneumonia		
	Partial clinical recovery (n = 85)	Complete clinical recovery (n = 1886)	*P*	Partial clinical recovery (n = 104	Complete clinical recovery (n = 835)	*P*
Age (years)						
<5	17 (20.0)	700 (37.1)	0.001	57 (54.8)	443 (52.7)	0.735
5–18	68 (80.0)	1,184 (62.8)		47 (45.2)	392 (47.0)	
Biological sex (male)	37 (43.5) n = 85	983 (52.2) n = 1884	0.119	54 (51.9)	439 (52.6)	0.900
Underlying disease or condition	14 (16.7) n = 84	367 (21.6) n = 1696	0.278	33 (32.4) n = 102	174 (21.3) n = 819	0.011
Cardiovascular disease	1 (6.7) n = 15	35 (8.4) n = 417	1.000	5 (16.1) n = 31	22 (11.5) n = 191	0.466
Gastrointestinal disease	0 (0.0)	0 (0.0)	NA	2 (2.0) n = 98	0 (0.0) n = 804	0.012
Genetic disease	0 (0.0) n = 15	6 (1.4) n = 411	1.000	1 (3.2) n = 31	8 (4.2) n = 189	1.000
Hematologic malignancy	0 (0.0) n = 15	17 (4.1) n = 411	1.000	0 (0.0) n = 31	6 (3.2) n = 189	0.598
History of preterm birth	1 (6.7) n = 15	10 (2.4) n = 410	0.330	5 (16.1) n = 31	10 (5.2) n = 191	0.025
Neurological disease	0 (0.0) n = 15	12 (2.9) n = 414	1.000	5 (14.7) n = 34	13 (6.9) n = 188	0.164
Obesity	2 (13.3) n = 15	39 (9.4) n = 417	0.644	3 (9.7) n = 31	19 (10.0) n = 191	1.000
Pulmonary disease	6 (40.0) n = 15	117 (27.0) n = 433	0.255	14 (42.4) n = 33	48 (24.1) n = 199	0.028
Renal disease	0 (0.0) n = 15	3 (0.7) n = 411	1.000	0 (0.0) n = 31	2 (1.1) n = 188	1.000
Rheumatological disease	0 (0.0) n = 15	3 (0.7) n = 410	1.000	0 (0.0) n = 31	1 (0.5) n = 188	1.000
Solid tumor	0 (0.0) n = 15	4 (1.0) n = 411	1.000	1 (3.2) n = 31	5 (2.7) n = 189	1.000
Transplantation	0 (0.0) n = 15	3 (0.7) n = 410	1.000	0 (0.0) n = 31	2 (1.1) n = 189	1.000
COVID-19 vaccination	4 (8.3) n = 48	36 (3.3) n = 36	0.080	1 (1.4) n = 71	7(1.2) n = 596	0.596
Predominant strain during infection^†^			<0.001			<0.001
Pre-delta/pre-omicron	25 (29.4)	347 (18.4)		8 (7.7)	195 (23.4)	
Delta	42 (49.4)	740 (39.2)		55 (52.9)	327 (39.2)	
Omicron	18 (21.2)	799 (42.4)		41 (39.4)	313 (37.5)	
Ct of SARS-CoV-2 RT-PCR, median (IQR)						
N gene	20.2 (17–24) n = 60	21.3 (17.6–26.5) n = 1306	0.078	21.5 (18.3–25.2) n = 87	21.4 (18.3–26.8) n = 656	0.769
*ORF* gene	22.2 (16.7–25) n = 30	22 (18.4–27.9) n = 945	0.400	20.8 (17.5–25) n = 68	21.1 (18.5–25.9) n = 550	0.458
Clinical severity						0.001
Mild	85 (100)	1,883 (100)	NA	-	-	
Moderate	-	-		89 (85.6)	751 (89.9)	
Severe	-	-		9 (8.7)	76 (9.1)	
Critical	-	-		6 (5.8)	8 (1.0)	
Laboratory results at COVID-19 diagnosis						
Anemia	11 (73.3) n = 15	253 (69.1) n = 366	1.000	12 (63.2) n = 19	99 (64.7) n = 153	0.894
Leukopenia	1 (6.7) n = 15	59 (16.2) n = 366	0.483	3 (15.8) n = 19	29 (18.8) n = 154	1.000
Neutropenia	1 (6.7) n = 15	37 (15.2) n = 243	0.705	3 (15.8) n = 19	20 (13.0) n = 154	0.722
Lymphopenia	2 (13.3) n = 15	64 (26.5) n = 242	0.367	3 (15.8) n = 19	46 (28.9) n = 154	0.282
Thrombocytopenia	0 (0.0) n = 15	8 (2.1) n = 371	1.000	3 (15.8) n = 19	2 (1.3) n = 151	0.010
Elevated AST	1 (16.7) n = 6	46 (18.7) n = 246	1.000	2 (28.6) n = 7	13 (25.5) n = 51	1.000
Elevated ALT	0 (0.0) n = 6	22 (8.9) n = 246	1.000	2 (28.6) n = 7	9 (18.0) n = 50	0.610
Elevated TB	0 (0.0) n = 4	3 (1.7) n = 172	1.000	1 (25.0) n = 4	0 (0.0) n = 42	0.087
Elevated DB	0 (0.0) n = 4	7 (4.1) n = 172	1.000	2 (50.0) n = 4	0 (0.0) n = 42	0.006
Hypoalbuminemia	0 (0.0) n = 4	2 (1.2) n = 172	1.000	1 (25.0) n = 4	3 (7.3) n = 41	0.320
CRP (mg/L), median (IQR)	3.4 (2.9–7.7) n = 5	1.6 (0.6–4.2) n = 117	0.080	5.2 (3.8–9.5) n = 7	3.0 (1.8–13.0) n = 36	0.377
Treatment						
FPV	28 (32.9)	997 (52.9)	<0.001	96 (92.3)	691 (82.8)	0.013
Steroids	0 (0.0)	27 (1.4)	0.628	8 (7.7)	63 (7.5)	0.957
Antibiotics	2 (2.4)	47 (2.5)	1.000	15 (14.4)	80 (9.6)	0.123
Antifungal agents	0 (0.0)	1 (0.1)	1.000	0 (0.0)	0 (0.0)	NA
Oxygenation	0 (0.0)	7 (0.4)	1.000	14 (13.5)	84 (10.1)	0.285
ICU admission	0 (0.0)	4 (0.2)	1.000	14 (13.5)	35 (4.2)	<0.001
Duration of ICU stay, median (IQR)	0 (0.0)	5 (3–8) n = 3	NA	3 (2.5–8) n = 8	7.5 (4.5–11) n = 28	0.073
Duration of FPV administration, median (IQR)	4 (4–5) n = 28		0.893	4 (4–5) n = 96	4 (4–5) n = 689	0.069
Time from diagnosis until FPV administration, median (IQR)	1 (1–2) n = 28	1 (0–1) n = 993	0.075	1 (0–2) n = 96	1 (0–2) n = 690	0.834
Time from COVID-19 symptom onset until FPV administration, median (IQR)	2 (1–3) n = 27	1 (1–3) n = 967	0.407	1 (1–3) n = 69	2 (1–3.5) n = 588	0.354

1 Mann–Whitney U or Student’s t-tests were used to compare continuous variables. Data presented as n (%) unless stated otherwise.

† The ancestral Wuhan strain and alpha variant were the predominant circulating strains during the first to third waves (Pre-delta/omicron); delta and omicron variants were the predominant circulating strains during the fourth and fifth waves, respectively.

1 ALT, alanine transaminase; AST, aspartate transaminase; COVID-19, coronavirus disease 2019; Ct, cycle threshold; CRP, C-reactive protein;

1 DB, direct bilirubin; FPV, favipiravir; ICU, intensive care unit; IQR, interquartile range; NA, not applicable; N gene, nucleocapsid gene; *ORF* gene, open reading frame gene; RT-PCR, reverse transcription polymerase chain reaction; SARS-CoV-2, severe acute respiratory syndrome coronavirus 2; SD, standard deviation; TB, total bilirubin.

**Supplementary Table 3.** Laboratory results at FPV treatment initiation and completion for patients with and without COVID-19 pneumonia

	COVID-19 non-pneumonia			COVID-19 pneumonia		
	Treatment initiation	Treatment completion	*P*	Treatment initiation	Treatment completion	*P*
Hematocrit, median (IQR)	36.5 (33.9–38.5) n = 120	36.6 (34.1–39) n = 750	0.569	36.7 (34.8–39.1) n = 537	0.493
Anemia	90 (75) n = 120	519 (69.3) n = 749	0.205	58 (60.4) n = 96	341 (63.5) n = 537	0.564
WBC (cells/mm^3^), median (IQR)	7,295 (5,210–9,345) n = 120	6,970 (5,380–9,260) n = 751	0.947	7,750 (6,145–9,775) n = 96	7,810 (5,900–9,950) n = 535	0.961
Leukopenia	24 (20) n = 120	151 (20) n = 750	0.973	17 (17.7) n = 96	86 (16.1) n = 535	0.690
ALC (cells/mm^3^), median (IQR)	1,539 (776–4;3,120) n = 93	2,444 (1,346–4,329) n = 666	<0.001	2,295 (1,475–4,556) n = 96	2,806 (1,616–4,681) n = 464	0.186
Lymphopenia	51 (54.8) n = 93	288 (43.3) n = 665	0.036	37 (38.5) n = 96	200 (37.5) n = 533	0.850
ANC (cells/mm^3^), median (IQR)	4,277 (2,280–6,497) n = 94	3,172 (1,946–5,096) n = 668	0.005	3,520 (2,110–5,661) n = 96	3,197 (1,981–5,200) n = 533	0.272
Neutropenia	8 (8.5) n = 94	106 (15.9) n = 667	0.060	11 (11.5) n = 96	85 (16) n = 533	0.260
Platelet × 103 (cells/mm^3^), median (IQR)	284 (223–326) n = 120	278 (226–337) n = 753	0.862	269 (236–333) n = 93	289 (230–356) n = 535	0.649
Thrombocytopenia	4 (3.3) n = 120	26 (3.5) n = 753	1.000	2 (2.2) n = 93	27 (5.1) n = 535	0.290
AST (U/L), median (IQR)	30 (22–37) n = 77	37 (27–47) n = 652	0.001	44 (27–49) n = 34	39 (30–50) n = 331	0.808
Elevated AST	12 (15.6) n = 77	191 (29.3) n = 651	0.011	8 (23.5) n = 34	115 (34.7) n = 331	0.188
ALT (U/L), median (IQR)	16 (12–21) n = 26	18 (13–25) n = 652	0.023	19 (16–25) n = 33	20 (14–29) n = 330	0.829
Elevated ALT	6 (7.8) n = 77	59 (9.1) n = 651	0.712	4 (12.1) n = 33	50 (15.2) n = 330	0.800
TB (mg/dL), median (IQR)	0.3 (0.2–0.4) n = 34	0.3 (0.2–0.4) n = 219	0.661	0.3 (0.2–0.4) n = 24	0.3 (0.2–0.5) n = 174	0.099
Elevated TB	2 (5.9) n = 34	5 (2.3) n = 219	0.239	1 (4.2) n = 24	3 (1.7) n = 174	0.406
DB (mg/dL), median (IQR)	0.1 (0.1–0.2) n = 32	0.1 (0.1–0.2) n = 211	0.735	0.1 (0.1–0.2) n = 23	0.1 (0.1–0.2) n = 170	0.107
Elevated DB	2 (5.9) n = 34	11 (5.0) n = 219	0.689	1 (4.2) n = 24	11 (6.3)n = 174	1.000
Albumin (g/L), median (IQR)	45 (42–47) n = 37	44 (41–47) n = 232	0.481	44 (42–46) n = 24	43 (40–46)–n = 188	0.105
Hypoalbuminemia	1 (2.7) n = 37	13 (5.6) n = 232	0.700	0 (0) n = 24	13 (6.9) n = 188	0.370

1 Mann–Whitney U or Student’s t-tests were used to compare continuous variables. Data presented as n (%) unless stated otherwise. Anemia, lymphopenia and thrombocytopenia, elevated AST, ALT, alkaline phosphatase, and albumin were determined according to age reference values [1].

1 ALC, absolute lymphocyte count; ALT, alanine transaminase; ANC, absolute neutrophil count; AST, aspartate transaminase; COVID-19, coronavirus disease 2019; DB, direct bilirubin; FPV, favipiravir; IQR, interquartile range; NA, not applicable; TB, total bilirubin; WBC, white blood cell.
